# Bis-biguanide dihydrochloride inhibits intracellular replication of *M. tuberculosis* and controls infection in mice

**DOI:** 10.1038/srep32725

**Published:** 2016-09-07

**Authors:** Hongbo Shen, Feifei Wang, Gucheng Zeng, Ling Shen, Han Cheng, Dan Huang, Richard Wang, Lijun Rong, Zheng W. Chen

**Affiliations:** 1 Unit of anti-tuberculosis immunity, CAS Key Laboratory of Molecular Virology and Immunology, Institute Pasteur of Shanghai, Chinese Academy of Sciences, Shanghai 200031, China; 2 Department of Medical Microbiology and Parasitology, Shanghai Medical College, Fudan University, Shanghai 200032, China; 3 Department of Microbiology, Zhongshan School of Medicine, Key Laboratory for Tropical Diseases Control of the Ministry of Education, Sun Yat-sen University, Guangzhou 510080, China; 4 Department of Microbiology/Immunology, and Center for Primate Biomedical Research, University of Illinois College of Medicine, 835 S. Wolcott Avenue, MC790 Chicago, IL 60612, United States; 5 Department of Microbiology and Immunology, University of Illinois College of Medicine, 835 S. Wolcott Avenue, MC790 Chicago, IL 60612, United States.; 6 CAS Institut Pasteur of Shanghai, Shanghai 200031, China

## Abstract

While there is an urgent need to develop new and effective drugs for treatment of tuberculosis (TB) and multi-drug resistant TB (MDR-TB), repurposing FDA (U.S. Food and Drug Administration) -approved drugs for development of anti-TB agents may decrease time and effort from bench to bedside. Here, we employed host cell-based high throughput screening (HTS) assay to screen and characterize FDA-approved, off-patent library drugs for anti-*Mycobacterium tuberculosis* (MTB) activities. The cell-based HTS allowed us to identify an anti-cancer drug of bis-biguanide dihydrochloride (BBD) as potent anti-mycobacteria agent. Further characterization showed that BBD could inhibit intracellular and extracellular growth of *M. smegmatis* and slow-growing *M. bovis* BCG. BBD also potently inhibited replication of clinically-isolated MTB and MDR-TB strains. The proof-of-concept study showed that BBD treatment of MTB-infected mice could significantly decrease CFU counts in the lung and spleen. Notably, comparative evaluation showed that MTB CFU counts in BBD-treated mice were lower than those in rifampicin-treated mice. No apparent BBD side effects were found in BBD-treated mice. Thus, our findings support further studies to develop BBD as a new and effective drug against TB and MDR-TB.

Tuberculosis (TB) remains one of the leading causes of global morbidity and mortality among infectious diseases largely due to HIV (Human Immunodeficiency Virus) pandemics and drug-resistance^1^. Multidrug-resistant TB (MDR-TB) is defined as an infection form of *M. tuberculosis* (MTB) resistant to at least two first-line anti-TB drugs: isoniazid (INH) and rifampicin (RIF)^2^. The 2014 WHO (World Health Organization) Global TB Report estimated that MDR-TB occurs in 3.5% and 20.5% of new and previously treated TB cases, respectively^3^ and about 480,000 MDR-TB cases were reported in 2013^3^. MDR-TB is quite difficult to treat, as the treatment is long and expensive, and often associated with high frequencies of adverse events and failure^4^. While MDR-TB poses a significant threat to global TB control^5^, there is an urgent need to develop new and effective anti-TB drugs.

Very few of the current anti-TB drugs can exert bactericidal effect on intracellular MTB^6^,^7^. MTB can survive and replicate in host cells, and exploit cellular shelters to alleviate or even avoid the killing by anti-TB drugs^8^,^9^. Moreover, the ability of MTB to undergo adaptive metabolic changes within the host may also affect drug activity and potency^10^,^11^. Rational drug development strategy should target both bacterial and host/cellular factors^12^,^13^ for developing novel drugs capable of inhibiting or killing intracellular MTB. Thus, host cell-based drug screening assay may serve as an ideal model system to select new anti-TB drug candidates against intracellular MTB.

We have employed mycobacteria-infected macrophages/lung epithelial cells for cell-based high-throughput screening (HTS), and screened and characterized FDA (U.S. Food and Drug Administration)-approved, off-patent library drugs for their abilities to inhibit/kill intracellular mycobacteria and MTB. This cell-based HTS allowed us to identify anti-mycobacterial agents capable of inhibiting intracellular bacilli. One of the leading anti-mycobacterial agents identified from FDA-approved compounds is an anti-cancer drug, bis-biguanide dihydrochloride (BBD)^14^,^15^. We found that BBD was able to inhibit growth of intracellular and extracellular mycobacteria including fast-growing *M. smegmatis*, slow-growing *M. bovis* BCG, as well as clinically-isolated MTB and MDR-TB strains. Furthermore, BBD treatment of *M. tuberculosis* H37Rv-infected mice could significantly decrease MTB CFU (Colony-Forming Units) counts in lungs and spleens, achieving more apparent therapeutic effect than RIF.

## Results

### Construction and optimization of human cell-based high-throughput screening assay

**Figure 1:**
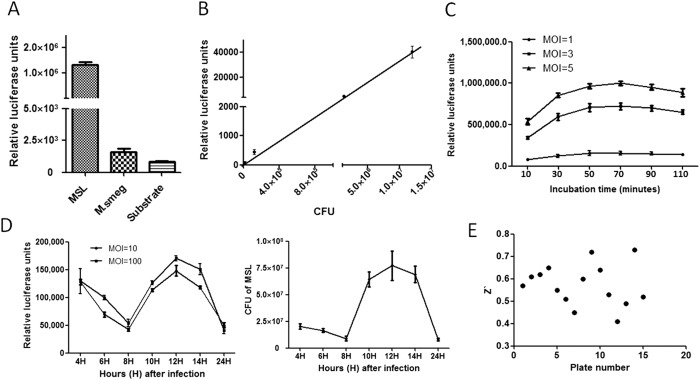
Construction and optimization of cell-based high throughput screening system. (**A**) Luciferase activities of positive MSL clones were measured by an EnVision plate reader. (**B**) Correlation between RLU and MSL CFU. (**C**) Optimization of incubation time of substrate with MSL infected A549 cells. (**D**) Growth dynamics of MSL in A549 cells. (**E**) The Z′ factors were calculated from signals of DMSO (Dimethyl Sulphoxide, solvent of drugs) and SM (Streptomycin, positive drug) wells and are plotted for 10 randomly selected plates.

To establish a robust high throughput screening (HTS) assay with a high ratio of signal to noise, we constructed a recombinant *M. smegmatis* strain expressing luciferase reporter gene and used it to infect human lung epithelial cell A549 and human macrophage THP-1 cells, respectively. To accomplish this, recombinant plasmid pMV261 contained luciferase reporter gene was transformed into *M. smegmatis* strain through electro-transformation method. Positive recombinant *M. smegmatis* clones expressing luciferase reporter gene (MSL) were characterized for luciferase coding gene by PCR (Polymerase Chain Reaction)-based sequencing method. Characterized MSL clones were further confirmed through luciferase activity assay. Results showed that positive MSL clones incubated with neolite luciferase substrate could give rise to ~10^3^-10^4^ folds of relative luciferase units (RLU) signals compared to MSL clones without substrate or substrate without MSL (Fig. 1A). To test the correlation between luciferase activity and the number of MSL strains, we simultaneously measured luciferase activities of different numbers of MSL bacteria and CFU counts of MSL on culture plates. As shown in Fig. 1B, luciferase activities (RLU values) increased proportionally over CFU counts of MSL, indicating the utility of our MSL reporter system for screening assay.

We then used MSL to infect A549 cells and measured RLU signals in microtiter plates. We found that MSL-infected A549 cells, once incubated with luciferase substrate, could also give more than 10^3^ folds of RLU signals (Mean ± SD, 1177407 ± 102305.6) compared to A549 cells with and without substrate (497.2 ± 133.9). Thus, these results demonstrated that MSL-infected A549 cells in microtiter plates could provide cell-based high throughput screening (HTS) system to screen new drug candidates against intracellular mycobacteria.

To increase the efficacy of this HTS assay system, we optimized incubation time. When comparing substrate RLU values obtained at different time points of MSL-infected cells, we found that 60 minutes of incubation was optimal and could be used in further experiments (Fig. 1C). We also found that changes in overall trends of RLU values derived from different MOIs (Multiplicity of Infection) were similar although absolute RLU values correlated with increases in MOIs (Fig. 1C).

To define the growth dynamics of live MSL in A549 cells, we comparatively tested RLU values and CFU at different time points after MSL infection of A549 cells. RLU values and CFU counts of MSL began to increase at ~8 hour and reached the peak at ~12 hour after MSL infection of A549 cells (Fig. 1D), suggesting that RLU values in this assay could mirror numbers of live MSL bacteria in host cells. This was indeed consistent with the intracellular growth dynamics of wild-type *M. smegmatis* as seen in CFU counts^16^. We therefore selected 12-hour as an optimal detection time for drug screening after MSL infection of A549 cells.

To facilitate identification of compounds inhibiting growth of intracellular mycobacteria, we added 10 μM gentamicin (GM) to the culture after MSL entry to cells. We found that GM was efficient to kill the remaining extracellular MSL in the culture as GM hardly killed intracellular MSL due to its limited ability to enter host cells (MIC of GM to intracellular MSL is about 20 μM, MIC to extracellular MSL is about 3 μM).

### High throughput screening (HTS) and identification of a bis-biguanide dihydrochloride as a drug candidate inhibiting intracellular mycobacteria growth

We then performed HTS assay screening drug compounds from Maybridge and Prestwick libraries in MSL-infected A549 cells and THP-1 cells, respectively. The inhibition ratio of isoniazide (INH), rifampicin (RIF) and streptomycin (SM) at 10 μg/ml on growth of MSL in A549 cells were tested, and they were 90.45 ± 1.73%, 70.78 ± 0.89% and 93.26 ± 2.04%, respectively. So, SM was included as a positive control. Z′ factor, a commonly-used parameter of screening quality, was employed to monitor HTS. Average Z′ factor for 10 randomly selected plates was about 0.57 ± 0.092 (mean ± STDEVA) (Fig. 1E), implicating an excellent quality of HTS.

To ensure the reliability or reproducibility of HTS, we performed two rounds of screenings, with each compound in duplicate in 384-well plates (Supplementary Figure 1A,B). In the first round screening, cut-off inhibitory hits were initially defined by mean RLU values-2SD derived from screening 384-well plates of MSL-infected A549 and THP-1 cells in duplicate experiments, respectively. Compounds/drugs identified from the first round of screening were confirmed for inhibition in the second-round screening in MSL-infected A549 or THP-1 cells (Supplementary Figure 1A,B).

**Figure 2:**
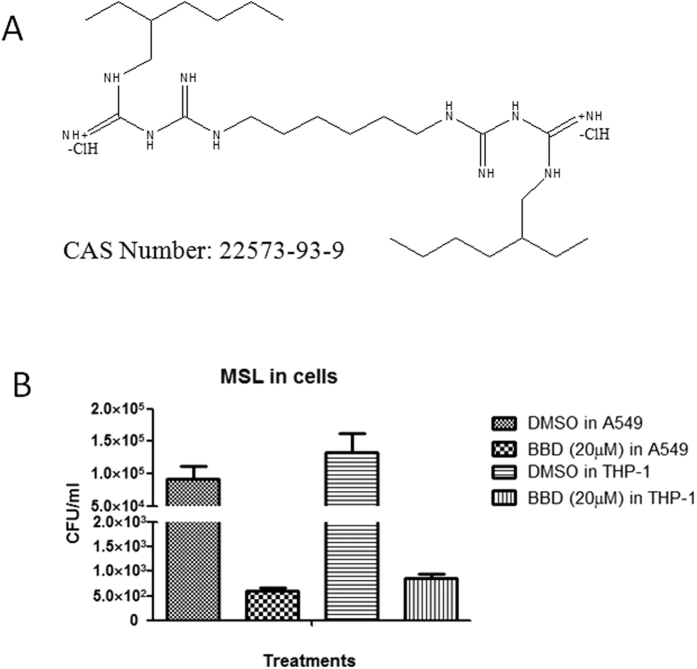
One novel anti-TB drug agent, bis-biguanide dihydrochloride (BBD). (**A**) Structure and Chemical Abstracts Service (CAS) number of BBD. (**B**) CFU counting results of MSL-infected THP-1 and A549 cells treated by DMSO and BBD (20 μM) for 12 hours, respectively.

The two rounds of screening allowed us to identify a promising drug candidate (code prestw-777) in well #K16 of No.3 plate of Prestwick library drugs. This FDA-approved drug, a bis-biguanide dihydrochloride (BBD), consistently exhibited MSL with very low RLU values in both two rounds of screening of MSL-infected A549 cells and THP-1 cells, respectively (Supplementary Figure 1A,B). Of note, BBD had chemical feature of a di-biguanide compound (Fig. 2A). To confirm the inhibition of intracellular MSL by BBD, we conducted CFU assay employing wild-type *M. smegmatis* infection of A549 and THP-1 cells in the presence of BBD at 20 μM (Fig. 2B). The CFU assay confirmed that BBD was able to inhibit intracellular *M. smegmatis* growth in host cells (Fig. 2B). While BBD was recently reported as a potent anti-cancer drug potentially interacting with DNA^14^,^15^, we demonstrated for the first time that BBD could also potently inhibit mycobacterial growth.

Furthermore, we conducted cytotoxicity studies of the IC50 for BBD in peripheral blood mononuclear cells from rodents using the CCK8 assay. Results show that IC50 is189.4 μg/ml. Such IC50 value is generally considered acceptable for further studies.

### BBD inhibited intracellular and extracellular growths of slow-growing mycobacteria, *M. bovis* BCG

**Figure 3:**
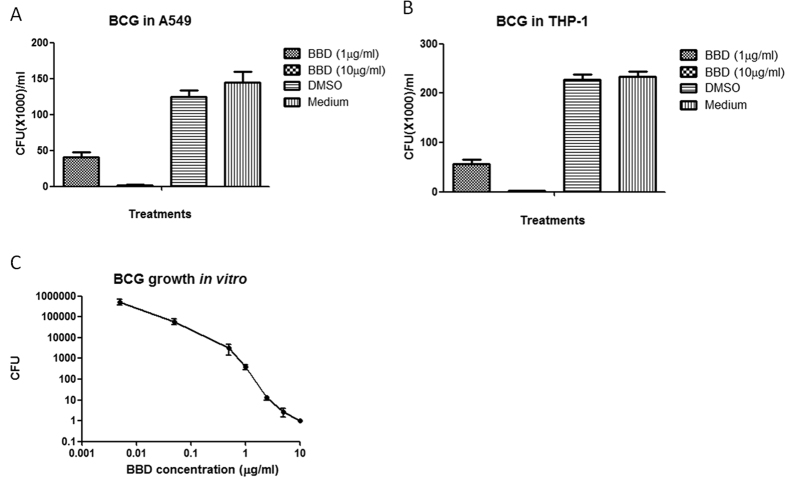
Effect of BBD on *M. bovis* BCG growth in cells and outside cells in bacterial culture medium. BBD can inhibit *M.bovis* BCG growth in A549 (**A**) or THP-1 cells (**B**) and outside host cells in bacterial culture medium 7H9 (**C**) and the inhibitory effects are in dose-dependent manner.

We then sought to determine if BBD could inhibit slow-growing mycobacteria, *M. bovis* BCG. We assessed BBD for the ability to inhibit intracellular BCG growth in host cells as well as extracellular BCG replication in bacterial culture medium. Thus, *M. bovis* BCG-infected human cells were incubated for three days with BBD at 1 μg/ml and 10 μg/ml, respectively, and lysate were counted for BCG CFU on 7H10 agar plates. Results showed that BBD could also significantly inhibit BCG growth both in A549 (Fig. 3A) and THP-1 (Fig. 3B) cells, respectively. In parallel, we examined if BBD could directly inhibit mycobacteria outside host cells in bacterial culture medium (*in vitro*). The results at day 3 showed that BBD could directly inhibit extracellular replication of BCG in bacterial culture medium in a dose-dependent manner (Fig. 3C). The minimal 99% inhibition concentration (MIC_99_) for BBD was about 0.05–0.1 μg/ml.

Therefore, BBD could inhibit intracellular BCG growth as well as extracellular BCG replication in bacterial culture medium.

### BBD remarkably inhibited replication of clinically isolated MTB and MDR-TB strains

The ability of BBD to inhibit *M. smegmatis* and *M. bovis* BCG raised a critical question as to whether BBD could also exert inhibitory effect on highly virulent MTB, the mycobacterium causing TB in humans. We assessed BBD for the capability to inhibit various MTB and MDR-TB strains isolated from TB patients (Table 1) using the standard BACTEC 960 detection system^17^. We found that BBD could remarkably inhibit growth of clinical isolates of drug sensitive MTB strains at a concentration as low as 0.05 μg/ml (Table 2). The inhibitory efficacy of BBD appeared to be similar to that of classical 1^st^ line drug RIF (Table 2). Promisingly, BBD could also inhibit the growth of clinically-isolated MDR-TB strains in concentrations of ≥0.05 μg/ml (Table 2), whereas RIF was not able to inhibit growth of these MDR-TB strains even at a high concentration of 10 μg/ml (Table 2). Thus, BBD could remarkably inhibit replication or growth of clinically isolated MTB and MDR-TB strains.

### A proof-of-concept (POC) study showed that BBD treatment of MTB-infected mice reduced MTB bacterial burdens in the lung and spleen

**Figure 4:**
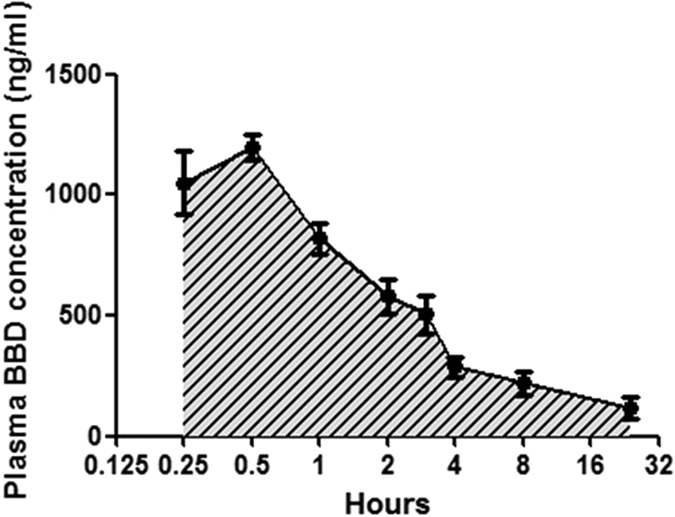
Plasma concentration of BBD detected by HPLC over time after IP injection of BBD in mice. Data are mean ± SD (standard deviation) from 4 mice.

**Figure 5:**
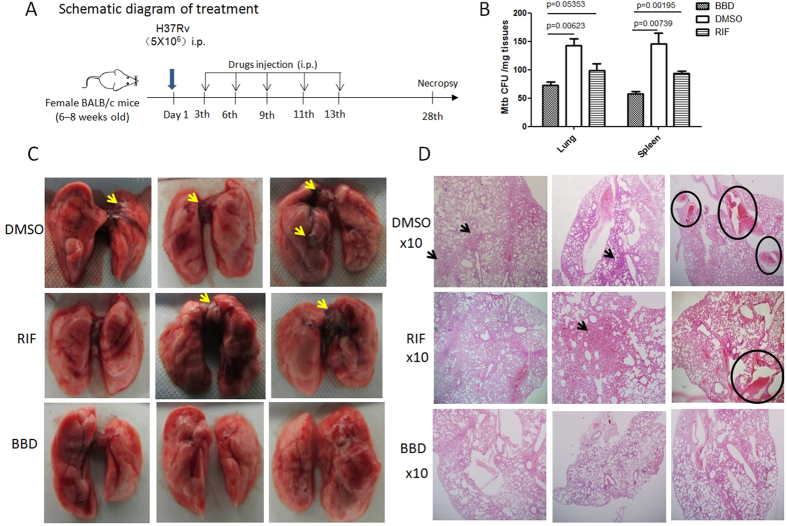
A proof-of-concept study showed that BBD treatments of MTB-infected mice reduced MTB bacterial burden and attenuated MTB-driven lesions in lungs. (**A**) Time line for MTB infection and BBD treatments in mice. (**B**) CFU counts of *M. tuberculosis* H37Rv in lung and spleen homogenates. Lung or spleen tissue homogenates generated from each mouse was serially diluted and used for MTB CFU enumeration (MTB CFU/mg tissue). (**C**) Digital photos of lung lobes from representative mice from PBS/DMSO-, RIF- and BBD-treated groups. Yellow arrow denotes TB lesions characterized by hemorrhages, swelling or exudative inflammation. (**D**) Representative hematoxylin-eosin (HE)-stained histopathological images (×10) of lung sections from PBS/DMSO-, RIF- and BBD-treated groups. Black arrow means consolidation or inflammatory exudates containing epithelioid cells and lymphocytes hyperplasia. Black circle means hemorrhages.

We then sought to evaluate BBD for therapeutic effects against MTB infection in mice infected with MTB H37Rv strain. Our pharmacokinetics (PK) studies demonstrated that intraperitoneal injection (IP) of mice with 0.125 mg BBD per 20 g·body weight could reach maximum plasma concentration of about 1200 ng/ml at 0.25–1 hr after IP injection (Fig. 4). These results suggested that treatment of mice with such BBD dose could reach a plasma concentration higher than MIC99 (0.05–0.1 μg/ml equals to 50–100 ng/ml) to inhibit/kill Mtb. Since this was a proof-of-concept (POC) study, MTB-infected mice were treated by intra-peritoneal (i.p.) injection of 0.125 mg BBD per 20 g·body weight every 3 days for a total of 5 doses (Fig. 5A). This pilot regimen for POC was selected based on the following measures: (i) effective doses of BBD in the *in vitro* experiments; (ii) PK data (Fig. 4) and mouse body weight/fluid volume; (iii) *in vivo* dosing of RIF treatment as RIF and BBD share comparable molecular weights.

Our POC regimen appeared to be safe as the toxicity experiments in uninfected normal mice demonstrated a lack of notable differences in body weights, complete blood counts and other routine parameters between BBD-treated and PBS-treated groups (data not shown). As controls, two groups of mice were treated with PBS/DMSO (Dimethyl Sulphoxide) and RIF at a dose higher than BBD, respectively. At 4 weeks after MTB infection, MTB CFU counts in the lung and spleen were measured and comparatively evaluated between groups.

BBD-treated group exhibited lower numbers of bacilli organisms in tissue homogenates of the lung and spleen than PBS/DMSO-treated group. Surprisingly, CFU counts in the spleen of BBD-treated mice were statistically lower than those of RIF-treated mice, although BBD dose (0.125 mg/20 g·bodyweight) was only a half of RIF (0.25 mg/20 g·bodyweight) (Fig. 5B). These results suggested that BBD treatments of MTB-infected mice in the POC study could reduce MTB bacterial burdens in the lung and spleen, and that BBD tentatively induced better therapeutic effect than RIF.

### BBD treatments of MTB-infected mice led to a decrease in MTB-driven lesions in the lung

Finally, we sought to determine whether BBD treatments of MTB-infected mice could attenuate pathological damage caused by MTB infection. Gross pathology analysis of mouse lung lobes showed that PBS/DMSO-treated mice exhibited apparent MTB-driven lesions such as unresolved hemorrhage, swelling or exudative inflammation (Fig. 5C). In contrast, these pathological changes were much milder in the lungs of BBD-treated and RIF-treated mice (Fig. 5C). Consistently, histopathological analysis showed that overall lung sections in BBD-treated mice displayed no apparent lesions or destruction of lung structures, whereas sections in PBS/DMSO-treated control mice exhibited hemorrhage and consolidation or inflammatory exudates containing epithelioid cells and lymphocytes hyperplasia (Fig. 5D). Lung sections of RIF-treated mice appeared to have milder lesions of pulmonary structures compared to PBS/DMSO (Fig. 5D). These results therefore suggest that BBD treatments of MTB-infected mice could attenuate MTB-induced lesions in lung.

## Discussion

In the current study, we have established a cell-based high throughput screening (HTS) assay to identify drug candidates capable of inhibiting intracellular mycobacteria. In this HTS system, human macrophages of THP-1 cells and lung epithelial cells of A549 serve as mycobacterium-infected cells since primary types of these cells are the main host targets for MTB bacilli infection^18^,^19^,^20^. We utilized MSL for infection and reporting of these host cells in the cell-based HTS, so that HTS assay could be done in BSL2 (Biosafety Shelter Laboratory, Level 2) lab without a special need for BSL3 operation. It is noteworthy that very few of infected cell-based HTS have been employed to identify new drug candidates capable of inhibiting intracellular MTB^21^,^22^,^23^, and that most HTS assays appear to be done using host-cell-free systems. To our knowledge, we are among the first to simultaneously use mycobacterium-infected macrophages and lung epithelia cells in parallel for cell-based high throughput screening of large libraries of FDA-approved drugs. We show that our cell-based HTS assay has acceptable reproducibility and fidelity. We also demonstrate the utility for our cell-based HTS assay as we successfully use it to identify and subsequently characterize an anti-cancer drug BBD for the ability to potently inhibit intracellular *M. smegmatis*, *M. bovis* BCG and clinically isolated MTB and MDR-TB strains.

Our data demonstrate that BBD can function as a promising anti-MTB drug candidate capable of inhibiting intracellular and extracellular replication of mycobacteria. The inhibition of intracellular mycobacteria by BBD is initially identified by cell-based MSL HTS assay and subsequently verified by CFU assay on cell lysate. Further studies using *M. bovis* BCG confirm that BBD can also directly inhibit or kill slow-growing BCG bacilli outside host cells in bacterial culture medium while also being able to inhibit intracellular BCG in host cells. It is likely that BBD may readily go through cell membrane and get into intracellular compartment for action against intracellular MTB. In fact, it has been shown that BBD functions as apoptosis-promoting anticancer agent and it also stimulates increased insulin secretion by β-cells in rat pancreatic islets^14^. Both therapeutic actions may somehow be explained by the pharmacological ability of BBD to penetrate membrane into cells. We anticipate that BBD might also inhibit intracellular MTB as it does for *M. smegmatis* and *M. bovis* BCG. This notion is also supported by the results from our therapeutic experiments in MTB-infected mice. The preliminary therapeutic study shows that CFU counts in lungs of BBD-treated mice are lower than RIF-treated animals. The presumption that BBD is better than RIF in getting inside cells may help to explain why the lower MTB burdens are seen in BBD-treated mice.

Our findings support the current efforts to repurpose clinical drugs for anti-TB agents. The development of an entirely new anti-TB drugs requires long and expensive processes for an arduous FDA approval^24^. Repurposing of FDA-approved drugs for development of anti-TB agents may decrease the time and efforts to develop novel anti-TB drugs from bench to bedside as these drugs are off-patent, with well-defined functions, mechanisms of action, pharmacological and toxicological properties^25^. We have identified BBD as potent anti-MTB agent by screening drugs from Prestwick Chemical Library, and subsequently characterized it in cultures and evaluated it for anti-MTB efficacy during treatments of MTB-infected mice. While further studies are needed to elucidate BBD-targeted/BBD-bound MTB protein(s) and inhibition mechanisms, the current findings provide a justification to conduct further studies for developing BBD as a new and effective anti-TB drug.

## Materials and Methods

### Cell lines, strains and plasmids

Human lung epithelial cell line A549 was cultured in Dulbecco’s modified Eagle’s medium (DMEM) supplemented with 10% heat-inactivated fetal bovine serum (FBS) at 37 °C under 5% CO_2_. Human macrophage THP-1 cells were cultured at RPMI 1640 containing 10% FBS, 10 mM HEPES, 2 mM glutamine.

*Escherichia coli* (*E. coli*) strains DH5α were cultured in Luria-Bertani (LB) medium broth and on agar supplemented with 25 μg/ml kanamycin. *M. Smegmatis* (ATCC 700084/mc(2)155) strains, *M. bovis* BCG Danish strain (ATCC 35733) and *M. tuberculosis* strains were grown in Middlebrook 7H9 broth and on Middlebrook 7H10 agar supplemented with 10% oleic acid-albumin-dextrose-catalase-enriched Middlebrook (OADC) according to our previously reported protocols^26^. *M. smegmatis* mc(2)155 and BCG Danish strain were obtained from ATCC. *M. tuberculosis* H37Rv and clinical isolates of *M. tuberculosis* were provided by Shanghai Pulmonary Hospital of China (Table 1). Plasmid of pMV261 was kept in our lab. Plasmid with luciferase gene was obtained from Dr. Rong Lijun lab in University of Illinois at Chicago^27^,^28^.

### Construction of recombinant *M. smegmatis* expressing luciferase (MSL) strains

To development a high-quality high-throughput screening (HTS) system, we have inserted luciferase encoding *luc* gene into an *E.coli-Mycobacteria* shuttle plasmid pMV261 and constructed recombinant plasmid pMV261-*luc*. Sequences of primers used to amply *luc* gene as following: PL1: 5′-CTGCAG(Pst I)ATGGAAGACGCCAAAAAC-3′; PL2: 5′-GTCGAC(Sal I)CAATTTGGACTTTCCGCCC-3′. The recombinant plasmids were transferred to *M. Smegmatis* mc(2)155 strain when they were isolated from *E.coli* DH5α. Parameters of electroporation were 1.8kv, 25uF, 200 Ω^29^. And the positive *M. Smegmatis*-luciferase clones (named MSL) were incubated with neolite luciferase substrate (PerkinElmer, USA) at room temperature for 60 minutes. Luciferase activity (relative luciferase units, RLU) was measured by an EnVision plate reader (PerkinElmer, USA)^30^.

### Correlation between RLU and the Colony-Forming Units (CFU) of MSL

30 μl of bacterial culture in serial dilutions of MSL at exponential growth phase was incubated with 30 μl neolite luciferase substrate for 60 minutes. Then, RLU values are measured by an EnVision plate reader. At the same time, bacteria were inoculated on 7H10 agar plates containing OADC after serial dilutions. Four weeks after cultivation, the bacilli CFU were counted.

### Optimization of screening parameters

A549 cells cultured in 75 cm flasks were infected by MSL with multiplicity of infection (MOI) of 50 for 2 hours, and then washed three times with PBS. After trypsinization, cells were purified using Ficoll-Paque Plus density gradient centrifugation to get rid of attached bacteria completely and then cultured in 384-well plates. To optimize the incubation time for substrate and MSL in cells, we have added neolite luciferase substrate and incubated for 10, 30, 50, 70, 90, 110 minutes before RLU measurement. To determine the growth dynamics of MSL in A549 cells, we have compared the RLUs of MSL growth in A549 cells for 4, 6, 8, 10, 12, 14, 24 hours after infection.

THP-1 cells were stimulated by 25 μg/ml Phorbol 12-myristate 13-acetate (PMA) for 24 hours, washed three times with PBS and rest for another 24 hours in fresh medium before MSL infection as protocol of infection in A549 cells.

### High-Throughput Screening assay

The Maybridge chemical library and the Prestwick drug library (commercialized by Prestwick Chemical, Illkirch, France) were used in this study. Three hundred twenty unique compounds were arrayed in a 384-well plate at a 10-mM concentration in Dimethyl Sulphoxide (DMSO), leaving columns 1, 2, 23, and 24 with DMSO or positive drugs of Streptomycin (SM).

MSL-infected A549 or THP-1 cells were seeded into flat-bottom, 384-well plates (Culture Plate; PerkinElmer, Waltham, MA) in a 30-μL assay medium containing 10 μM GM (keep to inhibit growth of extracellular MSL) using JANUS liquid handler MDT (Modular Dispense Technology; PerkinElmer). And 0.2 μL of each compound was added per well in 384-well plate through a pin tool (V&P Scientific, San Diego, CA) and mixed thoroughly using a JANUS liquid handler MDT. This resulted in a final concentration of 20 μM (0.25% DMSO) for all compounds. After incubation at 37 °C, 5% CO_2_, with high humidity for 12 hours, 30 μL of neolite luciferase substrate (PerkinElmer, Waltham, MA) was added to each well, and plates were incubated at room temperature for 60 minutes before RLU measurement.

The RLU value with a reduction of mean RLU-2SD (standard deviation) value at every 384-well plate was taken as the criterion for designating a compound as a “hit”. The “hits” in both MSL infected A549 and THP-1 cells with twice repetition experiments in each cell line were then cherry-picked into 384-well plates and take part in the second round screening. In the second round screening, 90% inhibition of RLU were taken as cutoff comparing to the average RLU values of negative controls (DMSO wells). The compounds or drugs identified as hits from two rounds of screening were subjected to bacterial CFU assay for inhibition of intracellular mycobacterial growth after wild-type *M. smegmatis* infection of A549 cells and THP-1 cells. Drugs or compounds capable of inhibiting *M. smegmatis* at ≥90% inhibition were selected for further characterization.

Z′ factor is the most commonly used statistical parameter to assess screen quality. The Z′ factor was calculated from the normalized signals from DMSO and SM control wells on 10 randomly selected plates with the following equation: 1 – 3 (StdDMSO + StdSM)/(MeanDMSO–MeanSM)^27^,^28^.

### Measurement of anti-mycobacterial drug activity outside host cells in mycobacterium culture medium

After two rounds screening in MSL infected A549 and THP-1 cells, respectively, “hits” were assessed for inhibition activities against extracellular mycobacteria including fast-growing *M. smegmatis*, slow-growing *M. bovis* BCG and clinical isolates of *M. tuberculosis* strains in bacterial culture medium in the presence drug concentrations of 10, 5, 2.5, 1, 0.5, 0.05, 0.005 μg/ml *in vitro*. After 3 days incubation of *M. smegmatis* or *M. bovis* BCG with drugs, culture medium of bacteria was diluted and cultured in 7H10 agar plates for 3–4 weeks. CFU of bacteria were counted.

The drug inhibitory activity in growth of clinically isolated *M. tuberculosis* strains were tested by BACTEC MGIT 960 culture system (BD company, USA)^31^. The bacterial suspensions were inoculated into new MGIT vials in duplicate with drugs in different concentrations. RIF was taken as parallel positive control drug. DMSO and medium were taken as negative control. The MGIT 960 culture was considered positive if the instrument signaled positive.

We conducted cytotoxicity studies of the IC50 for BBD in peripheral blood mononuclear cells from rodents using the CCK8 assay. Briefly, mouse blood were collected and peripheral blood mononuclear cells (PBMC) were isolated from EDTA-treated blood using Ficoll-Paque Plus density gradient centrifugation. PBMC were then cultured with RPMI1640 media supplemented with 10% FBS (Invitrogen). Cells were cultured in 96-well plate at 3 millions/ml with BBD at different concentrations for 24 hours. Then, cell viability was evaluated by the Cell Counting Kit-8 assay (Beyotime Biotechnology) according to the manufacturer’s guidelines. The half-maximal inhibitory concentration (IC50) values were calculated from dose-response curves utilizing Prism.

### Therapeutic treatment experiments after MTB infection of mice

Female Balb/c mice (6–8 weeks old) were challenged by intra-peritoneal (i.p.) injection with 5 × 10^6^ CFU *M. tuberculosis* H37Rv per mice to establish acute MTB infection model. Infected mice were randomly divided into three groups as follows: Group 1, mice treated with PBS/DMSO (control group) by i.p.; Group 2, mice treated with RIF at 0.25 mg/20 g·mouse by i.p.; Group 3, mice treated with bis-biguanide dihydrochloride (BBD) at 0.125 mg/20 g·mouse by i.p. Each group consisted of 15 mice and treated every 3 days for 5 times starting at day 3 after MTB infection (Fig. 4A). Four weeks after infection, mice were sacrificed and bacterial loads in lungs and spleens were determined by CFU counts. The *in vivo* studies were repeated twice.

This study was operated in ABSL-3 Laboratory of Sun Yat-sen University, China. All mice experiments were performed in accordance with recommendations in the Sun Yat-sen University Research Council Guide for Care and Use of Laboratory Animals. Animal study protocols were also reviewed and approved by the Sun Yat-sen University Institutional Animal Care and Use Committee.

### Histopathology analysis

Lungs of each mouse were excised and thoroughly evaluated for gross pathology. Lungs were then cut into two pieces; one was subjected to CFU counting, and the other was fixed in 4% neutral-buffered paraformaldehyde solution for 24 h. Then, lung tissue was embedded with paraffin. Series of sections with a thickness of 4–7 μm were then cut and stained with hematoxylin and eosin by standard methods, and images were obtained using a microscope (BX51WI; Olympus) and a digital camera (DP30BW; Olympus). Double blind analysis was then made by board certified pathologists and more than 10 slides of each lung were evaluated.

### Pharmacokinetics

The pharmacokinetics study was performed in mice. Mice were injected BBD at 0.125 mg/20 g·mouse by i.p. And plasma was collected from blood of 4 mice at 0.25, 0.5, 1, 2, 3, 4, 8, 24 h after drug injection, respectively. BBD plasma levels were determined using a validated liquid chromatographpay with tandem mass spectrometry assay. Concentrations of BBD in human plasma were evaluated using a non-compartmental approach by least squares (LS) linear regression analysis of the terminal phase of the semilogarithmic concentration-time curve. PK parameters were calculated according to standard methods using Kinetica software, version 4.2 (Thermo Scientific, USA).

### Statistical analysis

Statistical analysis was performed using SPSS Statistics 17.0 for Windows software package. Results were subject to independent samples t test, and P < 0.05 was considered statistically significant.

## Additional Information

**How to cite this article**: Shen, H. *et al*. Bis-biguanide dihydrochloride inhibits intracellular replication of *M. tuberculosis* and controls infection in mice. *Sci. Rep.* **6**, 32725; doi: 10.1038/srep32725 (2016).

## Supplementary Material

Supplementary Information

## Figures and Tables

**Table 1:** Information of clinical isolated mycobacteria strains.

Strain ID	Resistance to Clinical anti-TB drugs						Identification of drug resistance
	INH^1^	RIF^2^	EMB^3^	SM^4^	OFX^5^	KM^6^	
HZ0606	R	R	S	S	S	S	MDR^7^
HZ0626	R	R	R	R	R	S	MDR
HZ2575	S	S	S	S	S	S	MTB
HZ2643	S	S	S	S	S	S	MTB

^1^isoniazid.

^2^rifampicin.

^3^ethambutol.

^4^streptomycin.

^5^ofloxacin.

^6^kanamycin.

^7^Multi-drug resistant.

**Table 2:** Times to detection results by BACTEC MGIT 960 system.

Clinical isolated MDR-TB strains of HZ0606 and HZ0626				Clinical isolated MTB strains of HZ2575 and HZ2643			
Drug-conc. (μg/ml)	Growth Unit	TTD	Tube Status	Drug-conc. (μg/ml)	Growth Unit	TTD	Tube Status
RIF-0	4336.5	2d23.5 h	+	RIF-0	2043	2d12.5 h	+
RIF-0.005	3868	2d21.5 h	+	RIF-0.005	3064.5	3d15.5 h	+
RIF-0.05	3980.5	2d21 h	+	RIF-0.05	0	6d22 h	−
RIF-0.5	3937	2d21 h	+	RIF-0.5	0	6d22 h	−
RIF-2.5	4180.5	2d22 h	+	RIF-2.5	0	6d22 h	−
RIF-5	3989.5	2d22 h	+	RIF-5	0	6d22 h	−
RIF-10	5000	2d23 h	+	RIF-10	0	6d22 h	−
BBD-0	4545	2d22 h	+	BBD-0	3173	2d18 h	+
BBD-0.005	588	5d0 h	+	BBD-0.005	3951	4d4 h	+
BBD-0.05	0	6d22 h	−	BBD-0.05	0	6d22 h	−
BBD-0.5	0	6d22 h	−	BBD-0.5	0	6d22 h	−
BBD-2.5	0	6d22 h	−	BBD-2.5	0	6d22 h	−
BBD-5	0	6d22 h	−	BBD-5	0	6d22 h	−
BBD-10	0	6d22 h	−	BBD-10	0	6d22 h	−
DMSO	4318	2d22 h	+	DMSO	2292.5	2d14 h	+
Medium	1586	3d5 h	+	Medium	2291	2d13 h	+
CK(No MTB)	0	6d22 h	−	CK(No MTB)	0	6d22 h	−

TTD: Time To Detection (days and hours), average values of triple experiments.

Tube status: +, there is alive MTB strains; −, no alive MTB strains.
