# Author Correction: Bis-biguanide dihydrochloride inhibits intracellular replication of *M. tuberculosis* and controls infection in mice

**DOI:** 10.1038/s41598-020-62902-2

**Published:** 2020-04-15

**Authors:** Hongbo Shen, Feifei Wang, Gucheng Zeng, Ling Shen, Han Cheng, Dan Huang, Richard Wang, Lijun Rong, Zheng W. Chen

**Affiliations:** 1 Unit of anti-tuberculosis immunity, CAS Key Laboratory of Molecular Virology and Immunology, Institute Pasteur of Shanghai, Chinese Academy of Sciences, Shanghai, 200031 China; 2 Department of Medical Microbiology and Parasitology, Shanghai Medical College, Fudan University, Shanghai, 200032 China; 3 Department of Microbiology, Zhongshan School of Medicine, Key Laboratory for Tropical Diseases Control of the Ministry of Education, Sun Yat-sen University, Guangzhou, 510080 China; 4 Department of Microbiology/Immunology, and Center for Primate Biomedical Research, University of Illinois College of Medicine, 835 S. Wolcott Avenue, MC790, Chicago, IL 60612 United States; 5 Department of Microbiology and Immunology, University of Illinois College of Medicine, 835 S. Wolcott Avenue, MC790, Chicago, IL 60612 United States

Correction to: *Scientific Reports* 10.1038/srep32725, published online 07 September 2016

In this Article, Zheng W. Chen is incorrectly affiliated with ‘CAS Institut Pasteur of Shanghai, Shanghai 200031, China’. The correct affiliation for Zheng W. Chen is listed below.

Department of Microbiology/Immunology, and Center for Primate Biomedical Research, University of Illinois College of Medicine, 835 S. Wolcott Avenue, MC790 Chicago, IL 60612, United States

