# Supplementary material for: Bis-biguanide dihydrochloride inhibits intracellular replication of M. tuberculosis and controls infection in mice
Source: Sci Rep. 2016 Sep 7;6:32725. doi: 10.1038/srep32725 (PMC5013693; doi:10.1038/srep32725)
Supplement: Supplementary Information [file srep32725-s1.pdf]

Bis-biguanide dihydrochloride inhibits intracellular replication of *M. tuberculosis* and controls infection in mice

Hongbo Shen[1†*], Feifei Wang[2†], Gucheng Zeng[3], Ling Shen[4], Han Cheng[5], Dan Huang[4], Richard Wang[4], Lijun Rong[5], Zheng W Chen[4]

[1]Unit of anti-tuberculosis immunity, CAS Key Laboratory of Molecular Virology and Immunology, Institute Pasteur of Shanghai, Chinese Academy of Sciences, Shanghai 200031, China

[2]Department of Medical Microbiology and Parasitology, Shanghai Medical College, Fudan University, Shanghai 200032, China

[3]Department of Microbiology, Zhongshan School of Medicine, Key Laboratory for Tropical Diseases Control of the Ministry of Education, Sun Yat-sen University, Guangzhou 510080, China

[4]Department of Microbiology/Immunology, and Center for Primate Biomedical Research, University of Illinois College of Medicine, 835 S. Wolcott Avenue, MC790 Chicago, IL 60612, United States

[5]Department of Microbiology and Immunology, University of Illinois College of Medicine

[*]Correspondence address: hbshen@ips.ac.cn

[†]These authors contributed equally to this work.

Supplementary Figure. Discovery process of novel anti-TB drug agent, bis-biguanide dihydrochloride (BBD). Shown were representative screening results (RLU values) of MSL-infected THP-1 (A) and A549 (B) cells treated by drugs of No.3 plate of Prestwick database in 384-well plates. SM, a positive control drug, was located from wells of A-23 to H-23 in plates. Drug BBD located in the K16 well (Marked with cross) in every plate.

A

	1	2	3	4	5	6	7	8	9	10	11	12	13	14	15	16	17	18	19	20	21	22	23	24
A	20800	17200	21600	20600	10400	19600	22200	21600	22400	23800	16200	22000	3600	18800	18600	69000	15600	20600	23200	22000	14000	22200	1160	21600
B	22800	22800	13400	18600	19800	20600	24400	17000	30000	23600	20600	16200	21600	20000	25000	74600	17000	14000	17400	19000	3000	16600	1240	21800
C	18200	20400	47000	21200	20000	33600	23000	20400	15400	25400	21200	19200	17400	20000	41600	23200	20800	21400	22400	18000	4400	3000	920	18200
D	19400	15600	2200	18800	16800	18400	19800	19200	29200	39800	17000	20200	21200	20400	23000	23400	21600	27200	12600	22000	21200	25000	1360	21200
E	15800	21200	23800	20200	19200	19000	23800	22600	33600	26600	16200	28200	13600	21200	21000	24000	20800	26400	20000	30000	20400	17800	1220	21200
F	18800	21200	19200	20600	20200	102800	19200	24400	19600	18600	21600	19000	31200	2400	15400	17200	16200	16000	20400	122600	18400	15000	1180	21600
G	22400	21800	19200	29800	22200	83000	8600	21400	19600	18600	20400	22000	8800	19800	16800	20800	25000	19400	18600	21000	21000	20800	1300	19400
H	23800	23400	18200	20000	17600	101200	22800	20600	15400	18800	22400	14200	7600	22400	17000	21800	20400	18200	19600	16200	20200	25600	1220	21000
I	20000	21600	21000	42800	21200	21600	16200	19600	18600	12800	20800	28200	1200	20600	15200	21600	17600	19800	23000	23600	21000	20800	21600	21600
J	20800	18800	24200	18000	16400	19200	18800	21600	9000	17000	18000	7000	36200	14800	20600	19400	18600	17800	13000	36200	40800	8400	20200	21800
K	22600	18000	30400	19800	12800	63400	23000	19000	24600	16800	19200	21200	20200	16800	15800	1200	16000	16000	14200	20800	13800	16200	22000	24000
L	20400	19000	7600	18600	19000	34200	14400	14400	21200	22200	12400	21600	25000	20800	16600	71600	6800	16600	21800	18400	22800	21400	22400	21000
M	21600	30800	11600	16600	18200	18800	17200	11200	15600	18600	1400	45400	16600	17800	15200	8800	1600	17600	29800	43200	24400	15600	22200	19800
N	17000	21200	22200	28200	5200	36000	15200	22600	21200	9000	10200	13200	37200	15400	20000	8200	19600	17000	19800	24000	71600	20400	21600	27200
O	32200	28400	20600	21200	23000	9400	21200	26200	23800	19200	19400	42000	67200	18000	16800	17600	29400	13800	21600	11400	25400	20200	22600	22200
P	23800	19800	17200	25400	21600	66200	17400	20400	25800	17200	8800	24200	17400	19200	20200	17000	23600	19800	92200	31000	25800	24200	22800	23600

B

	1	2	3	4	5	6	7	8	9	10	11	12	13	14	15	16	17	18	19	20	21	22	23	24
A	11760	10240	8960	10840	1640	12040	11800	10240	12720	11600	13660	11500	2740	12940	10700	10020	7360	12260	11100	12980	9500	13220	320	13120
B	12080	11420	9720	10820	11660	12220	12160	10080	14000	12260	11620	7080	8500	9620	10800	17080	11280	5320	11480	11280	7780	13240	340	11720
C	12060	13260	25560	10460	12400	14440	13000	12140	11620	11980	10080	7160	11040	11260	8940	12080	12720	13640	15980	13720	4160	11860	320	14080
D	12860	11940	580	12640	11340	11460	12120	13140	15080	20300	12280	12160	13880	12680	13780	12940	12180	12900	8020	13560	14140	13280	360	12380
E	10940	11520	11820	13360	12940	13200	11860	10340	6840	17060	10740	18340	5940	13360	12760	11780	12520	16280	14020	18100	14180	12080	340	11920
F	11680	13280	12700	13300	13120	53540	12500	12620	11680	11960	12200	10340	18980	820	13160	12160	12820	8460	12900	35320	9300	8820	420	12840
G	12680	13180	8440	12880	11720	29720	6220	5480	12720	11380	11780	12860	3000	7760	13680	11600	15100	27200	13700	13640	13360	12800	360	13800
H	11620	11040	8920	14380	8780	37380	12220	11820	15420	10920	12120	9440	3340	6520	12960	14200	13760	10960	12260	9620	7920	13480	480	14180
I	12600	12600	12260	16940	11420	11560	13220	12540	12420	10900	10020	16300	520	14460	11560	11900	13940	12640	13880	13580	12500	14620	13100	13660
J	13240	11560	9600	12260	6760	9960	13560	11880	8580	12200	12520	3080	19340	11740	12340	12340	13060	10240	5720	19080	16940	3340	13120	13840
K	13360	12740	15900	12240	8660	22780	5020	9780	11920	12640	10600	13820	13460	14080	11480	380	13100	3640	6100	14800	8180	11900	12560	12100
L	12220	12500	1380	11640	8660	11500	11360	11040	15820	16160	8040	11300	15500	12960	10780	19940	4460	11680	11980	13980	13620	13500	13080	11680
M	12240	13580	7780	6840	11120	9600	8880	11700	10120	12600	640	13100	4200	14200	8600	5920	540	12180	18220	10000	13300	12000	13700	12060
N	11080	13040	12160	10580	2700	14280	11460	14380	11740	7800	9160	4240	14180	11940	13080	11340	13400	11660	12620	13780	21560	12780	13080	13040
O	12400	10960	11540	12480	10620	7360	13360	3360	12220	13080	10500	24340	25940	10460	9460	5340	12820	8940	11060	9340	12900	12880	11640	13000
P	12340	11120	11680	11660	12800	2480	15880	13900	11540	7920	2560	14840	9120	8780	12560	12240	13440	13240	61160	11040	12480	14440	12120	12380
